# Nonlinear continuum growth model of multiscale reliefs as applied to rigorous analysis of multilayer short-wave scattering intensity. I. Gratings

**DOI:** 10.1107/S0021889813012387

**Published:** 2013-06-07

**Authors:** Leonid Goray, Maxim Lubov

**Affiliations:** a Academic University, St Petersburg, 194021, Russian Federation; b Institute for Analytical Instrumentation RAS, St Petersburg, 194021, Russian Federation; c Ioffe Institute, St Petersburg, 195221, Russian Federation

**Keywords:** continuum growth model, boundary profiles, boundary integral equation method, scattering intensity, diffraction grating efficiency, multilayer relief gratings, soft-X-ray and extreme ultraviolet range

## Abstract

It is shown that taking into proper account certain terms in the nonlinear continuum equation of thin-film growth makes it applicable to the simulation of the surface of multilayer gratings with large boundary profile heights and/or gradient jumps. The proposed model describes smoothing and displacement of Mo/Si and Al/Zr boundaries of gratings grown on Si substrates with a blazed groove profile by magnetron sputtering and ion-beam deposition. Computer simulation of the growth of multilayer Mo/Si and Al/Zr gratings has been conducted. Absolute diffraction efficiencies of Mo/Si and Al/Zr gratings in the extreme UV range have been found within the framework of boundary integral equations applied to the calculated boundary profiles. It has been demonstrated that the integrated approach to the calculation of boundary profiles and of the intensity of short-wave scattering by multilayer gratings developed here opens up a way to perform studies comparable in accuracy to measurements with synchrotron radiation, at least for known materials and growth techniques.

## Abstract 2

The nonlinear continuum equation of thin-film growth is applicable to simulate the surface of multilayer gratings with large boundary profile heights and/or gradient jumps. The integrated approach to the calculation of boundary profiles and the intensity of short-wave scattering by multilayer gratings is a way of performing studies comparable in accuracy to measurements with synchrotron radiation for known materials and growth techniques.

## 1. Introduction

Recent progress in fabrication of multilayer X-ray diffraction gratings with boundaries of a specified shape and subatomic roughness stems primarily from the holographic and lithographic techniques employed in their manufacture, as well as from the advances achieved in materials chemistry, and the considerable steps forward accomplished in vacuum technology and methods developed for preparation and processing of Si plates and in nanometrology. The urgent need to continue the relevant research is made obvious by the pressing demands for development of novel high-resolution and efficient components of optical and electronic instrumentation in such areas as 6.X nm lithography, X-ray free-electron lasers (XFELs), resonant inelastic X-ray scattering, soft X-ray and extreme ultraviolet (EUV) astrophysics, X-ray microscopy *etc*. Comparison of the scattering intensities measured with sources of synchrotron radiation (SR) and XFELs with the results of calculations based on rigorous methods becomes an ever more pressing issue for short-wave optics.

It should be noted that in quantitative investigation of the evolution of thin-film boundary profiles one widely accepts microscopic methods, primarily transmission electron microscopy (TEM), atomic force microscopy (AFM) and near-field scanning optical microscopy. The first of these is fairly expensive and destructive, while the other two do not allow determination of inner layer boundary profiles of the sample prepared. Besides, microscopic methods are applicable only to studies of local characteristics of the structure formed.

One of the most universal approaches to investigation of the layer morphology and composition is based on reflectometry (scatterometry), including its short-wave version, which permits one to determine with a high precision and in an integral way the characteristics of the nanorelief of practically any thin-film material (Pietsch *et al.*, 2004 ▶). Of particular importance for the solution of ill-conditioned and nonuniquely solvable inverse problems in reflectometry (Goray, 2011 ▶) are the availability of (1) a universal and rigorous method for solution of the direct problem and (2) adequate information on the retrieved relief and/or refractive indices of the relevant materials for use as a starting approximation. Application of a modified method of boundary integral equations (MIM) (Goray *et al.*, 2006 ▶; Goray, 2010*a* ▶,*b* ▶) to analysis of the effect boundary profiles of gratings and mirrors with complicated rough interfaces produce on the X-ray scattering intensity is a novel approach based on the optical theory of continuous media, *i.e.* on the solution of Maxwell’s equations involving rigorous boundary conditions and radiation conditions. The MIM equations revealed that the intensities of X-ray scattering at boundaries with periodic and random asperities of the relief may differ considerably (by a few times) from the values derived with the use of various approximate models. It was found that this method operates equally well with nano-roughnesses of any kind and shape (Goray, 2009 ▶) that obey arbitrary statistics of distribution (not necessarily periodic or Gaussian). The present study focuses on the most essential differences of the MIM from the other available methods of boundary integral equations, as well as on the specific features of its application to the analysis of the intensity of short-wave scattering by mirrors and gratings. For a comprehensive description of the MIM the above references should be consulted.

In calculation of the intensity of X-ray scattering by multilayer gratings, one customarily resorts to layer boundary profiles obtained in scaling an initial to the final profile in the framework of various relevant models (Voronov, Anderson, Cambie, Gullikson *et al.*, 2011 ▶; Peverini *et al.*, 2007 ▶; Stearns *et al.*, 1998 ▶). Quite frequently, these calculations are not based on accurate information even on the initial or final boundary profile, which markedly complicates fitting the profiles of inner boundaries (Goray & Seely, 2002 ▶). This approach also does not permit one to account for (i) source noise, a factor that significantly influences the growth process, (ii) displacement of the large-scale grating profile initiated by profile smoothing or a change of the angle at which material is deposited on the grating, and (iii) variation of the surface relaxation parameters in the course of growth.

In the present study, to simulate the evolution of a thin-film profile, the continuum approach was employed because, in contrast to the discrete and dynamic methods, it provides the possibility of calculating the relief evolution over large temporal, ∼10^3^ s, and spatial, ∼10^2^ µm, scales. Besides, the continuum model permits one to study directly how the source noise and various nonlinear and geometric effects influence the growth process. Thus, for complex profiled surfaces, such as, for instance, randomly rough multilayer X-ray−EUV diffraction gratings, we believed it reasonable to choose a complex theoretical approach, which largely substitutes for experiment and permits one to calculate both the evolution of the boundary profile of layers and their optical response by short-wave reflectometry. The goal pursued by this study includes a theoretical investigation and computer simulation of boundary growth and of the intensity of short-wave scattering by multilayer gratings, which draw from the method proposed by us. In the rigorous calculations of the diffraction efficiency η(#) in the #-th order of multilayer gratings one makes use, for the first time, of the boundary profiles obtained by simulation of the growth of their layers. We have proposed and investigated the continuum equation describing the evolution of the profile of both the small-scale (random nano-roughness) and the large-scale (grating) relief.

This paper is organized as follows. §2 presents the model of growth of thin films on profiled surfaces which is based on the continuum approach developed for the purpose, taking into account the nonlinear terms of the kinetic equation. §3 outlines briefly a rigorous theory of scattering from multilayer gratings with arbitrary random noise. §4 deals with the theoretical efficiency results obtained for multilayer sawtooth gratings with simulated Mo/Si and Al/Zr boundaries and compares them with measurements performed on an SR source.

## 2. Model of thin-film growth on profiled surfaces

In the context of the proposed continuum approach, the variation of the film profile (boundary) height *h* with time *t* at point **r** on the surface is described by a kinetic equation taking into account various physical processes (Pellicione & Lu, 2007 ▶).

### 2.1. Mechanisms of growth and relaxation (smoothing) of a thin-film profile

Evolution of the boundary profile of a growing thin film is governed by two processes, to wit, deposition which translates into an increase of the profile height reckoned from the initial level, *h*_o_, and relaxation which smoothes asperities on the film surface. Surface relaxation is driven by the system (thin film) tending to attain in the course of growth a thermodynamic state in which its chemical potential would be the same at all points, **r**, of the surface. Among the main relaxation mechanisms are surface diffusion, evaporation–condensation and bulk diffusion (Pellicione & Lu, 2007 ▶; Mullins, 1957 ▶). For a given material system, the main mechanism of relaxation on the surface is governed by the growth conditions (substrate temperature, rate of deposition *etc*.), because the rates of diffusion and evaporation depend on the temperature and concentration of adatoms on the substrate. Migration barriers for the diffusion of atoms in the bulk of a film are higher than those on its surface and therefore bulk diffusion affects the variation of the film profile much less than surface diffusion does. Hence, for the sake of simplicity we are going to disregard bulk diffusion in what follows.

We assume the surface to be isotropic and two dimensional, in other words, that *h* can be represented by a function of coordinate *x* and time *t*. In the simplest case, the rate of height variation ∂*h*(*x*, *t*)/∂*t* in relaxation by the evaporation–condensation mechanism can be written in the form (after Mullins, 1957 ▶)
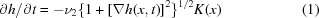
and, if relaxation occurs by the diffusion mechanism,
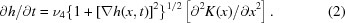
In equations (1) and (2), ν_2_ and ν_4_ are parameters defining the rates of the evaporation/condensation and the diffusion processes, respectively, and *K*(*x*) is the local surface curvature.

The actual form of an equation intended to describe the profile variation with time depends on the physical processes occurring at the surface of a film and in its bulk. In particular, if the substrate surface has steps of different height which exchange atoms, one should use other expressions for the diffusion terms. In a general case, the equation for the profile evolution can be written as
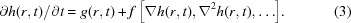
Here *g*(*r*, *t*) is the stochastic function defining the flux of atoms onto the film surface. While the actual form of the *g*(*r*, *t*) function depends on the type of the source, in most cases one can, however, assume the value of the atom flux to fluctuate about the average value 〈*g*(*r*, *t*)〉 = *I*_0_, with the flux fluctuations *I* (the noise) being uncorrelated:
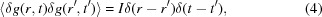
where Δ is Dirac’s delta function. The function *f* in equation (3) governs relaxation of the film surface and represents actually a sum of linear, 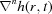, and nonlinear, 
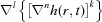
, terms, where *l*, *k*, *n* are positive integers. In each particular case, the specific form of *f* depends on the actual physical processes at work in the system.

We next consider the growth of multilayer gratings for use in X-ray optics in the framework of the continuum approach.

### 2.2. Kinetic model and simulation of the growth of rough multilayer gratings employed in short-wave optics

Fabrication of multilayer gratings for X-ray–EUV optics applications requires close control of the shape and roughness both of the lower and the upper boundaries and of the interface boundaries separating the layers. The intensity of short-wave scattering from such multilayer structures is influenced both by the small-scale relief created by source noise and the substrate not being perfect and by the large-scale pattern of groove facets. Therefore in a theoretical study of the evolution that the boundary profiles undergo in multilayer gratings particular attention should be focused on an exact numerical simulation of the growth process to properly take into account the above features.

The angle ϕ of the working face in typical high-frequency gratings with a blazed facet which are employed in short-wave optics is a few degrees, and the opposite angle β, at the base of the triangle, several tens of degrees. In this case, the equation for evolution of the surface profile for the two mechanisms of surface relaxation [see equations (1) and (2)] can be written as
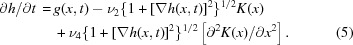
Simulation of the process of multilayer grating growth should take properly into account not only local curvature but the angle of incidence of the atomic (ion) beam as well. This is because the grating groove geometry may produce nonuniform deposition of material on the substrate as a result of shadowing effects (Voronov *et al.*, 2010 ▶). To reduce the effect of nonuniform deposition on the process of growth, the angle between the incident atomic beam and a growing grating groove facet should be close to 90°. In real growth equipment, however, it is extremely difficult to attain optimal deposition angles. Therefore in order to reduce the shadowing effects as much as possible, one resorts to predominant deposition on the nonworking facet of the groove, which is inclined to the substrate plane at a significantly larger angle.

Let us calculate now deposition flows striking a blazed profile assuming the atom beam to strike the substrate plane at an angle α, with β > ϕ. To leave the working facet unshadowed, the condition α > ϕ accepted in practice should be met. This condition translates for the number of atoms deposited on the working facet, disregarding noise, into
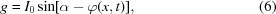
where ϕ(*x*, *t*) is the inclination of the working facet at the point with coordinate *x* at time *t*, ϕ(*x*, *t*) = arctan(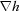). Then for the flux of atoms onto the nonworking facet we obtain
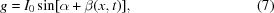
where β(*x*, *t*) is the inclination of the nonworking facet at point *x* at time *t*, β(*x*, *t*) = arctan(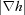).

Another significant factor capable of affecting noticeably the roughness of multilayer gratings is the difference in the magnitude of the relaxation parameters for the materials forming the layers. To cite an example, surface diffusion of material 1 over the layer made of material 2 may be considerably lower than that of material 2 over the surface of a layer of material 1. Note also that the relaxation parameters may vary in the course of growth; indeed, the near-surface layer of the growing grating may become heated under magnetron sputtering, an effect bringing about variation of the diffusion coefficients and, hence, of the relaxation parameters as well. Besides, large-scale roughness may end up in shadowing of the neighboring parts of the surface, thus altering the process of growth of the film in its immediate vicinity. One could mention here other physical chemical processes as well, whose account may be found necessary in the particular conditions of the technology and equipment employed.

## 3. Rigorous theory of scattering from multilayer randomly rough gratings

The MIM theory will be outlined here necessarily briefly, because its main parts, including specific aspects of a rigorous solution of short-wave diffraction problems (with a small ratio of wavelength λ to grating period *d* or correlation length ξ), are discussed in considerable detail by Goray and co-workers (Goray *et al.*, 2006 ▶; Goray, 2010*a* ▶,*b* ▶). The electromagnetic formulation of the problem of diffraction by a grating represented by an infinite periodic structure reduces to a system of Helmholtz equations for the *z* components of the electric and magnetic field in 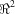, whose solution is quasi-periodic in the *x* direction, is described by radiation conditions in the *y* direction and satisfies rigorous boundary conditions at the interfaces between different materials. A multilayer grating can have a fairly large number of boundaries, up to a few thousand for applications in the hard X-ray range. In the case of classical diffraction, when the wavevector of the incident wave is perpendicular to the *z* direction, the system breaks up into two independent problems for the two main polarization states, whereas for conical diffraction the boundary values of the *z* components of the fields and their normal and tangential derivatives are coupled (Goray & Schmidt, 2010 ▶, 2012 ▶). The grating diffracts the incident wave into a finite number of outgoing plane waves, the so-called reflected and, possibly, transmitted modes (orders). The *PCGrate-SX* (v.6.5, http://www.pcgrate.com) program computes the energies of the orders and absorption for an arbitrary number of layers with boundaries of various types, including polygonal ones derived from measurements or growth simulation.

The effect of random roughness scattering on the grating efficiency can be rigorously taken into account with a model in which an uneven surface is represented by a grating with a large *d*, which includes an appropriate number of random asperities. *PCGrate* analyzes the complex structures which, while being multilayer gratings from a mathematical viewpoint, are actually rough surfaces for *d* >> ξ. If ξ ≃ λ and the number of orders is large, the continuous angular distribution of the energy reflected from randomly rough boundaries can be described by a discrete distribution η(#) of a grating (Goray, 2010*a* ▶). A MIM study of the scattering intensity starts with obtaining statistical realizations of profile boundaries of the structure to be analyzed, after which one calculates the intensity for each realization, to end with the intensity averaged out over all realizations. By selecting large enough samples, one comes eventually to properly averaged properties of the rough surface; however, this approach does not involve approximations, including averaging by the Monte Carlo method. The more general case of doubly periodic gratings (three-dimensional surfaces) may be considered in a similar way or by expressing the solution of the three-dimensional Helmholtz equation through solutions of the two-dimensional equation described below, an approach which may be resorted to in some cases (Goray, 2011 ▶).

Numerical solution of the two-dimensional Helmholtz equation by any rigorous method is known to involve difficulties for small λ/*d*. While the boundary integral equation method (Rathsfeld *et al.*, 2006 ▶) is usually stable, reliable and efficient, it is characterized by poor convergence and loss of accuracy in the short-wave range because quadrature calculations involve accumulation of errors in the orders. Increasing the matrix size and improving the calculation accuracy, as well as application of methods aimed at convergence acceleration, which are known to operate efficiently in the long- and medium-wave ranges, end up imposing unreasonably rigid requirements on computer time and memory for analysis of gratings in the X-ray and EUV ranges. In the case of computation of boundaries with a fine structure and depth *h* >> λ these requirements become still more demanding, particularly for gratings with a large number of layers. At the same time, the conventional integral method involves at least one collocation point per λ (the number of collocation points *N* specified for one period is the main parameter defining the accuracy of a method; Goray, 2010*a* ▶), whereas MIM operates fast and reliably for *N*λ/*d* << 1 in short waves. Thus, for instance, for *N* = 1000 and λ/*d* = 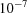, MIM uses only 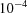 points per λ. In this case, however, the effective boundary depth *h*cosθ (θ is the angle of incidence on the grating reckoned from the substrate normal), the multilayer coating period Δ and λ should be of the same order of magnitude, at which the efficiency reaches high values in a specific order. This conclusion is applicable also both to echelles operating at any λ and to gratings designed for use over a longer-wave range (Goray, 2010*b* ▶).

## 4. Simulation of the multilayer grating boundary growth and of the efficiency

Drawing from the theoretical approaches proposed in §§2 and 3, we are passing on now to a study of the growth of layers of multilayer gratings to demonstrate the effect of boundary topology in a continuum film on spectral efficiencies. Diffraction gratings on blazed-profile Si substrates are fabricated by interference or electron lithography and selective etching in KOH of Si plates cut at an angle ϕ (Voronov *et al.*, 2010 ▶; Voronov, Anderson, Cambie, Cabrini *et al.*, 2011 ▶; Voronov, Gawlitza *et al.*, 2012 ▶; Voronov, Anderson *et al.*, 2012 ▶). While fabrication of gratings of a large size with *d* ≤ 200 nm for the short-wave range shows considerable promise, realization of the available design potential requires that the relevant technology produces a grating profile close to the ideal triangular geometry and coatings made up of tens or hundreds of atomic layers with subatomically smooth boundaries. Profile smoothing, an effect which becomes manifest in deposition of multilayer coatings and is of crucial importance for short-period gratings, is an essential factor in the problem of reducing η. Progressing to ever shorter periods with *d* ≤ 200 nm is impossible without first determining the effect boundary parameters exert on the efficiency, the easiest way to which lies through simulation of boundary growth and scattering intensity.

The growth of multilayer blazed-profile gratings was studied as applied to magnetron and ion-beam sputtering. Let us consider a two-dimensional problem, which appears only natural for classical gratings with a cylindrical groove geometry in space. The first step consisted in simulation of the process of growth for a multilayer grating realized on an Si substrate with a close-to-blazed groove profile. The relaxation parameters of the model of growth were derived from a least-squares comparison of the results of measurement with those obtained from simulation of the upper boundary profiles of gratings [see equations (1), (2) and (5)]. Next, the profile boundaries thus obtained were used as input data in calculation of the intensities of short-wave scattering. The results of the calculations of η(#) were compared with experimental data on scattering intensity and, whenever needed, the growth model parameters were refined. Numerical experiments were performed for multilayer gratings of two types, Mo/Si and Al/Zr. For analysis of the profile evolution we consider the universal equation (5) because, as has been demonstrated in the conjugate paper devoted to mirrors (Goray & Lubov, 2013 ▶), on the one hand, it describes well the growth of Al/Zr mirrors, while on the other, it allows adequately for local surface roughness, in other words, it offers a possibility to simulate the growth of a large-scale grating relief.

### 4.1. Mo/Si grating

We simulated a grating with *d* = 136 nm, ϕ = 6° and 30 pairs of Mo/Si coating layers, for which a record high η(+2) = 0.288 was measured in the SR beam with λ = 13.6 nm and θ = 11° (Voronov, Gawlitza *et al.*, 2012 ▶). The Mo/Si coating prepared by ion-beam deposition had Δ = 7 nm, and the ratio of the Mo layer thickness to Δ is Γ_Mo_ = 0.45. To study the effect of the substrate and of the relaxation parameters (5) on the profile variation of growing boundaries, we used averaged AFM measurements with 137 points of the Si substrate and upper boundary profiles of the multilayer grating. To take into account adequately the noticeable transformation of the boundary profiles during deposition of a multilayer coating and to attain the required accuracy of the solution of equation (5), the growth simulation program made use of the following parameters: *I*_0_ = 0.6 nm s^−1^, ν_2_(Mo) = 0.3 nm s^−1^, ν_4_(Mo) = 0.5 nm^3^ s^−1^, ν_2_(Si) = 0.3 nm s^−1^ and ν_4_(Si) = 1.5 nm^3^ s^−1^. The model deposition rate corresponded to the average deposition rate in the experiment (Voronov, Gawlitza *et al.*, 2012 ▶). Relaxation parameters ν_2_ and ν_4_ were chosen in such a way as to reproduce the final (upper) boundary profile of the multilayer grating (Voronov, Anderson, Cambie, Gullikson *et al.*, 2011 ▶) and to achieve the best coincidence of the experimental and calculated efficiencies.

It was assumed that the flow of atoms strikes the grating vertically. The refraction indices for Mo and Si were taken from Henke *et al.* (1993 ▶) (http://henke.lbl.gov/optical_constants/). To take into account random roughness and interdiffusion, in this particular example we employed Debye–Waller-type amplitude factors (σ_Si–Mo_ = 0.4 nm, σ_Mo–Si_ = 1.2 nm) similar to those used for plane interfaces (Pietsch *et al.*, 2004 ▶; Goray, 2010*b* ▶).

**Figure 1:**
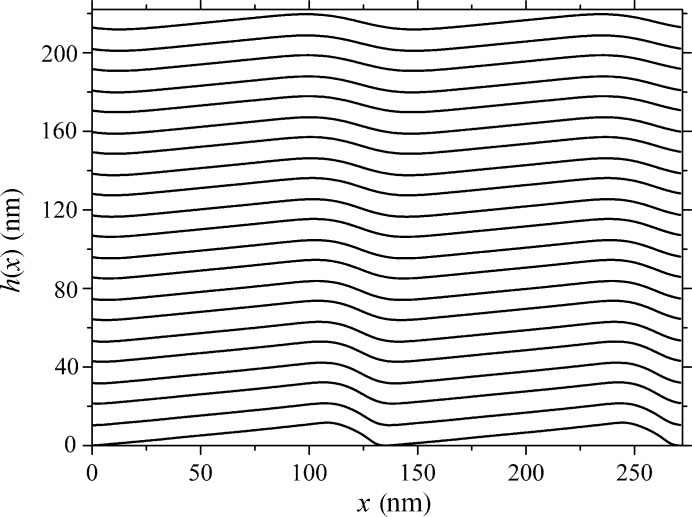
Boundary profiles of a grating with a period of 136 nm, blaze angle of 6° and 30 pairs of Mo/Si coating layers with period Δ = 7 nm. See §4.1 for the growth parameters used. For the sake of convenience, not all of the boundary profiles are presented.

**Figure 2:**
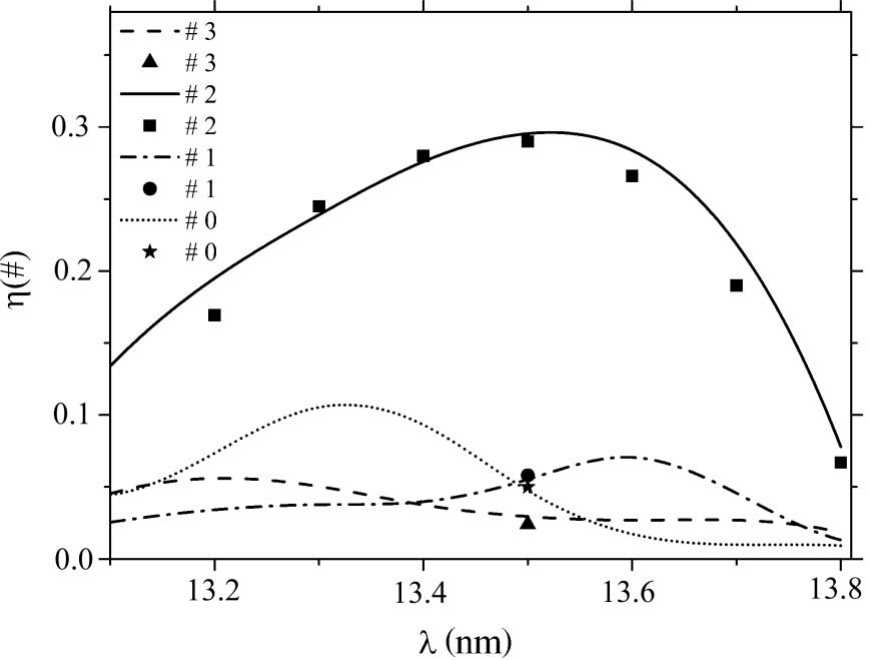
Spectral efficiency of grating orders with parameters as specified for Fig. 1 ▶ plotted for a radiation incidence angle of 11° near the wavelength of 13.5 nm: lines – present calculations; markers – measurements with SR [after Voronov, Gawlitza *et al.* (2012 ▶) and courtesy of LBNL].

The results of the calculations of the layer boundary profiles conducted with the given parameters and presented graphically in Fig. 1 ▶ revealed a certain smoothing of the profile top and groove, with the profile itself shifting to the left. This result is in a good agreement with the experimental data. The rigorous model of η calculation, combined with an approximate account of the effect of random boundary roughness, demonstrate a good agreement of the efficiency in the main diffraction orders with an experiment performed on the SR beam in the wave range under study, 13.1–13.8 nm (Fig. 2 ▶). The efficiencies obtained at other incidence angles likewise were found to match pretty well, which lends credence to the multilayer grating growth model chosen. A more accurate correlation of the results of measurements with efficiency calculations performed throughout the operating wavelength and incidence angle ranges can be performed by refining the boundary model, as well as by a rigorous account of the random roughness contribution. The results of the above calculations reveal a good convergence, and to simulate η of a grating with piecewise-linear boundaries with an accuracy of not worse than 0.01%, which was estimated from the energy balance, one would need *N* = 200 per boundary. The time expended to calculate one point is ∼40 s of operation on a low-end workstation with two Quad-Core Intel Xeon processors operating at a clock frequency of 2.66 GHz, a bus clock frequency of 1333 MHz, and with 16 GB of RAM.

### 4.2. Al/Zr grating

The most difficult to simulate is an EUV grating with *d* = 100 nm, ϕ = 6° and 20 pairs of Al/Zr layers deposited by magnetron sputtering. A comparison of calculated with measured reflection coefficients of a multilayer mirror (witness) revealed that the parameters Γ_Zr_ = 0.4 and Δ = 10.43 nm, incorporated in a model of multilayer Al/Zr grating coating, correlate well with the growth values and that the average interface roughness can be defined by σ ≃ 0.9 nm (σ_0_ ≃ 0.4 nm, ξ_0_ = 10 nm) (Voronov, Gawlitza *et al.*, 2012 ▶; Voronov, Anderson *et al.*, 2012 ▶). The data obtained in this comparison argue for ∼90% TE-polarized incident radiation (intensity).

**Figure 3:**
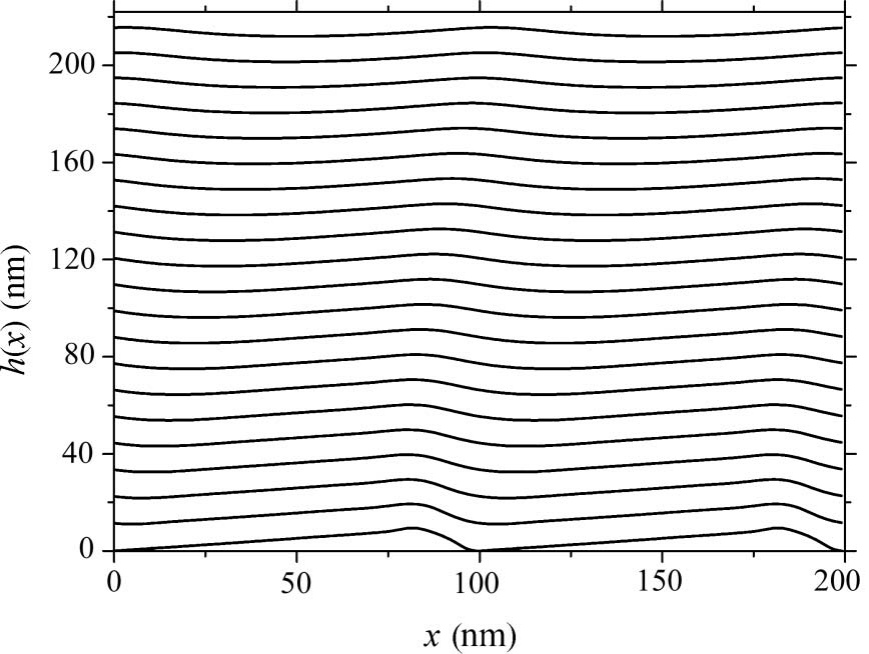
Boundary profiles of a grating with a period 100 nm, blaze angle 6° and 20 pairs of Al/Zr coating layers. See §4.2 for the growth parameters used. For the sake of convenience, not all of the boundary profiles are presented.

To study the influence exerted by the substrate, relaxation parameters [see equation (5)] and deposition conditions on variation of the profile of growing boundaries, we resorted to averaged AFM measurements of the profiles of the Si grating substrate and of the upper boundary of a multilayer grating with ten periods and 1000 points. Data obtained in AFM and TEM measurements were taken from Voronov, Gawlitza *et al.* (2012 ▶) and Voronov, Anderson *et al.* (2012 ▶). Al/Zr gratings exhibited a specific feature of growth in that they underwent a noticeable transformation of the boundary profiles, to wit, a decrease of the profile height (by about three times for the upper boundary), intense smoothing and a shift of the profile top to the right (Fig. 3 ▶). This variation of the profile is in marked contrast to that of Mo/Si gratings, whose profile varies substantially less during growth and shifts to the left (Fig. 1 ▶). This can be traced to the inclined geometry of the target material deposition employed in the relevant equipment to prevent shadowing (Voronov *et al.*, 2010 ▶). Simulation of the growth of Al/Zr gratings suggests that the shift of the profile top to the right results from the nonuniformity of the material flows deposited onto the working and nonworking facets of the blazed profile, which is caused by the atomic beam striking the substrate plane at an angle other than 90° (see §2.2). Significant smoothing of the boundary profile and a shift of the profile top to the right was observed to occur in simulation of growth conducted with the following parameters: *I*_0_ = 0.6 nm s^−1^, ν_2_(Zr) = 0.2 nm s^−1^, ν_4_(Zr) = 5.0 nm^3^ s^−1^, ν_2_(Al) = 0.22 nm s^−1^, ν_4_(Al) = 7.0 nm^3^ s^−1^, α = 78°. Growth parameters were chosen in a similar way as for the Mo/Si grating, taking into account additionally an inclination of the deposition flux from the substrate normal [see §2.2, equations (6) and (7)].

A rigorous approach employed to take into account the contribution arising from random roughness incorporated in the growth model brings about a decrease in the order efficiency, which was determined by means of *PCGrate* in the approach described in §3.

The refraction indices for Al were taken from Palik (1985 ▶) and those for Zr from Henke *et al.* (1993 ▶) because the relevant data for Zr are not given by Palik (1985 ▶). As established earlier (Seely *et al.*, 2004 ▶; Le Guen *et al.*, 2011 ▶), in the wavelength range of interest, 17–22 nm, the refraction indices of some materials derived from the approach developed in Henke *et al.* (1993 ▶) may be not accurate enough.

**Figure 4:**
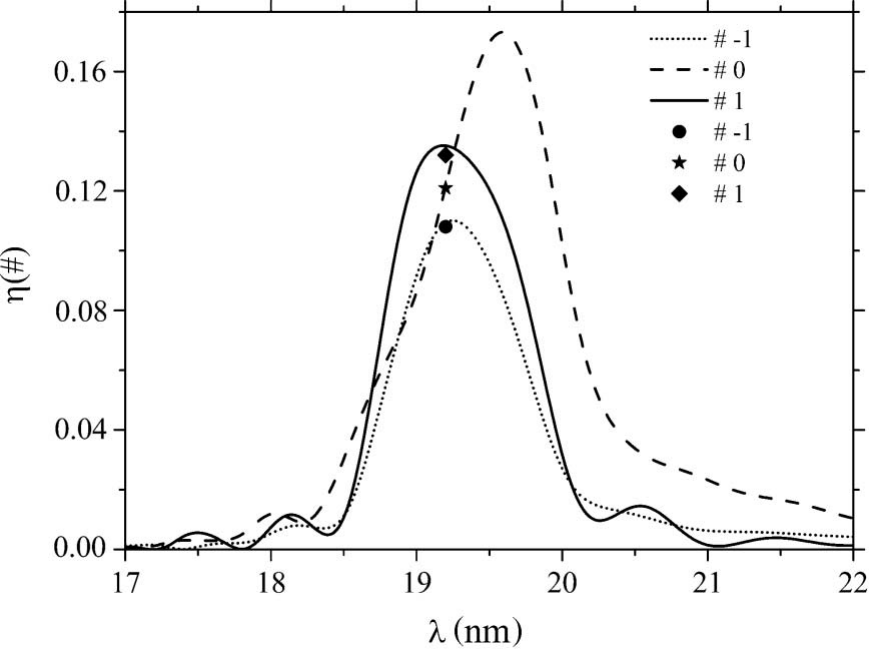
Spectral efficiency of grating orders with parameters as specified for Fig. 3 ▶, plotted for a radiation incidence angle of 11°: lines – present calculations; markers – measurements with SR [after Voronov, Anderson, Cambie, Gullikson *et al.* (2011 ▶)].

The complex grating model, taking into account the effects of source nonuniformity, the growth kinetics of deep asymmetrical boundaries with large gradients, and the random roughness of boundaries with varied r.m.s. and correlation lengths, demonstrates very good correlation of η in the main diffraction orders with the data obtained on the SR source for a number of incidence angles and wavelengths (Fig. 4 ▶). For the extreme case (not shown) measured at θ = 36° and λ = 17.2 nm, the discrepancy between the values of efficiency measured for the main diffraction orders by Voronov, Anderson, Cambie, Cabrini *et al.* (2011 ▶) and those obtained in our simulation is not over 5%. The match revealed for other θ and λ likewise turned out to be quite good, particularly if one takes into account the complexity of the boundary model employed, the intermixing between layers and the absence of reliable values for the refractive indices of Zr in the wavelength range studied. As for the calculations discussed here, the results exhibit good convergence, with *N* = 400 required for simulation of η of a grating with polygonal, randomly rough boundaries, with an error of ∼0.01% evaluated from energy balance considerations. The time required for the calculation of efficiencies for one λ is about five minutes on the workstation mentioned.

## 5. Conclusion

This study has been the first to demonstrate that correct simulation of the growth of boundaries in multilayer gratings with a large height and jumps in the profile gradient requires precise knowledge of the local surface curvature and of the nonuniform pattern of deposition of the material on the substrate. The efficient algorithms and the potential, fed into the vector electromagnetic code *PCGrate*, made it possible to study, with a standard PC, diffraction gratings with the use of data collected in simulation of boundary profile growth, and to obtain theoretical results offering the possibility of predicting the X-ray and EUV efficiency with an accuracy (within a few %) competing with that typical of measurements performed with SR for chosen samples.

It is worth noting that a good agreement between calculated and experimental values of spectral efficiencies of different diffraction orders obtained at different incidence angles can be achieved if, and only if, all of the simulated boundary profiles match perfectly with the experimental ones. Since electromagnetic computations of the efficiencies are performed within a widely proved rigorous method and compared with accurate measurements using SR, it can be concluded that the developed continuum model is also correct and allows one to fit a growth process adequately. The proposed numerical simulation permits one to radically cut the cost of technological processes and measurements on multilayer diffraction gratings with a desired boundary surface structure, an approach aimed at reaching values of η close to the theoretical limit.

The boundary integral equation method developed for analysis of the intensity of short-wave scattering by multilayer diffraction gratings can also be applied with considerable efficiency in studies of various gratings designed to operate in other spectral ranges, photonic crystals, Fresnel zone plates and rough mirrors. The model describing growth of multilayer films can be successfully used, in its turn, in studies of the growth process in semiconductor structures, more specifically, superlattices, buffer layers, low-dimensional nanostructures *etc*. The studies described here can be readily employed in designing high-resolution instruments for X-ray spectroscopy of the Sun and of other cosmic objects, research in the fields of plasma physics, X-ray lithography, correlation and resonance inelastic X-ray spectroscopy, and so on.
